# Are other fluorescent tags used instead of ethidium bromide safer?

**DOI:** 10.1186/2008-2231-21-71

**Published:** 2013-12-19

**Authors:** Soodabeh Saeidnia, Mohammad Abdollahi

**Affiliations:** 1 Medicinal Plants Research Center, Faculty of Pharmacy, Tehran University of Medical Sciences, Tehran 1417614411, Iran; 2 College of Pharmacy and Nutrition, University of Saskatchewan, Saskatoon, Canada; 3 Department of Toxicology and Pharmacology, Faculty of Pharmacy and Pharmaceutical Sciences Research Center, Tehran University of Medical Sciences, Tehran 1417614411, Iran

**Keywords:** Ethidium bromide, Carcinogenicity, Trypanocidal, Mutagenicity, Molecular biology

## Abstract

Ethidium bromide (EtBr) is a well-known fluorescent tag usually applied in molecular biological techniques like agarose gel electrophoresis. The mechanism of action for such compounds is known, in which these compounds are able to bind to the kinetoplastid DNA and to alter their conformation to Z-DNA molecules that stop replication of kinetoplastid DNA leading to *Trypanosoma* death. Although the usual amounts used in laboratories are considered as below the level required to cause toxicity (LD_50_ in oral administration in rat is 1.5 g/Kg), the mentioned concentrations are high enough to involve in replication of mitochondrial DNA in some human cell lines. Regarding the points that EtBr is very stable in the environment and if degraded especially by use of bleaches that result in formation of mutagenic compounds, there is a big concern about its use. Although application of EtBr is going to be restricted and replaced with other tags such as SYBR^®^ products, the safety of the new substituted compounds are still in doubt and except a few data, there is no essential evidence available to confirm that they are safer than EtBr. Further investigations are recommended to compare their relative biosafety hazards.

## 

Ethidium bromide (EtBr) is a well-known fluorescent tag (Figure 1) that is usually applied in molecular biological techniques. Agarose gel electrophoresis is one of them (Figure 2), in that EtBr is generally used. This compound acts as an intercalating agent to stain nucleic acids by marked florescent (orange) during exposure to UV light [1]. In addition to the wide application of this compound in molecular biology labs, it has been extensively applied in pharmacology labs to create a model of spinal cord demyelination in rats [2]. Furthermore, it has been employed since 1950 in veterinary medicine in order to therapy of trypanosomiasis in cattle via reduction of mitochondrial DNA copy numbers in proliferating cells [3]. Although this compound has been no longer used due to trypanocidal resistance, its related isometamidium chloride analogue has been applied instead. The mechanism of action for such compounds is to bind to the kinetoplastid DNA and to alter its conformation to Z-DNA molecules that stop replication of kinetoplastid DNA and result in *Trypanosoma* death [4].

**Figure 1:**
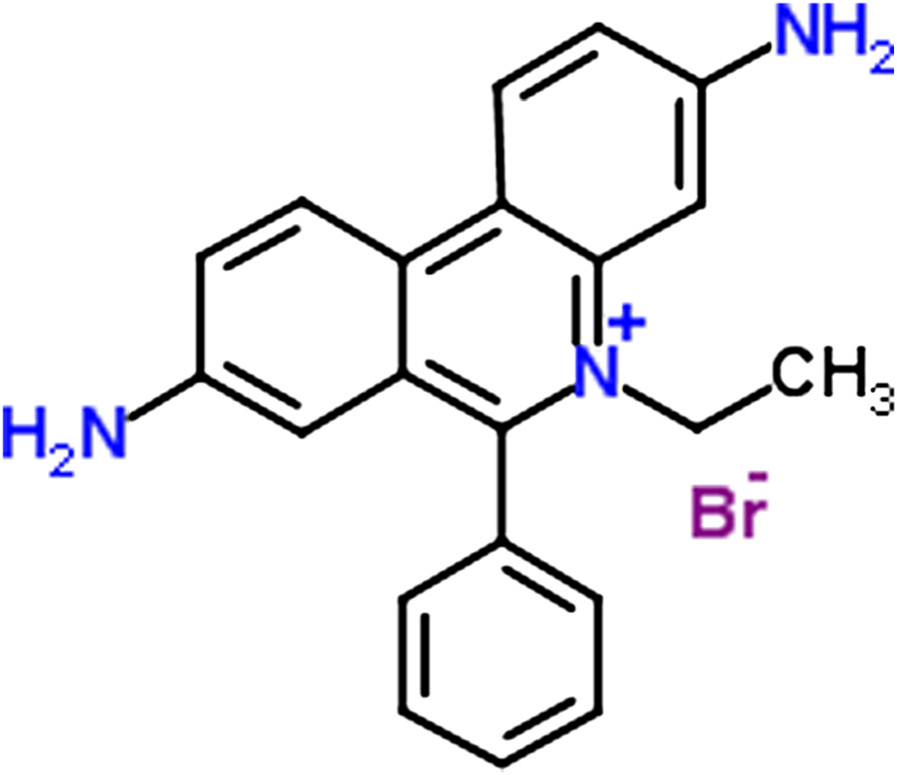
Chemical structure of ethidium bromide.

**Figure 2:**
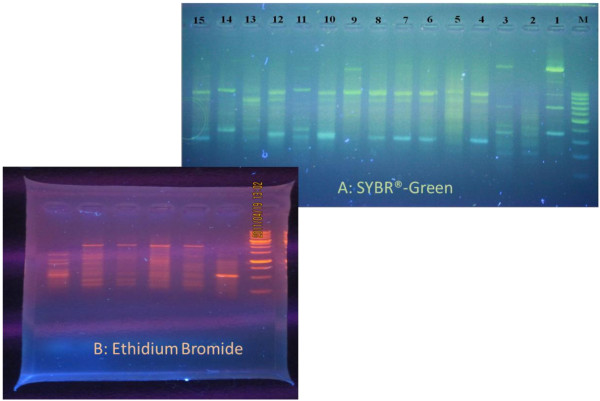
***In-house *** **photos (MPRC, TUMS) of gel agarose electrophoresis of DNA samples (*Phlomis *** **spp.) staining with SYBR**^**®**^**-Green (A) and *Polygonum *** **spp. with EtBr (B).**

With no doubt just by knowing the mode of intercalating DNA, EtBr is considered as a serious biohazard due to its mutagenicity, carcinogenicity and teratogenicity potentials depending on the organism exposed and the circumstances of exposure. It can be absorbed through the skin, and can irritate the eyes, mouth, and upper respiratory tract. If EtBr is used extensively in the lab, it can definitely contaminate a boundless work area. Therefore, the lab spaces should be decontaminated that burden extra expenses because techniques for managing EtBr waste are too costly.

Despites the serious toxicity of EtBr, it is still used in some labs because it is considerably less expensive in comparison to other compounds like SYBR®-based dyes (an asymmetrical cyanine dye used as a nucleic acid stain). Although the seller companies claim on safety of SYBR®-Green (Green Viewer), there are reports on higher mutagenicity of this compound in comparison to EtBr in the bacterial cells exposed to UV [5]. This retains the debate in place.

However, the question is “Is EtBr a potent carcinogen?” As a matter of fact, it should not be considered much dangerous because of its long history of application in treatment of animals’ trypanosomiasis. On one hand, the usual amounts used in laboratories are 0.25 to one microgram per mL considered as below the level required for toxicity (LD_50_ in oral administration in rat is 1.5 g/Kg). Of course, the mentioned concentrations are high enough to involve in replication of mitochondrial DNA in some human cell lines [6,7]. In a report on genetic toxicity, EtBr was classified as nonmutagenic in rats and mice and thus the fear of using this chemical is unjustified. However, they confirmed that EtBr powder is extremely hazardous and its use should be stopped. It is found that mutagenicity of EtBr does not happen when hepatic microsomes do not exist. This means that EtBr itself is not directly mutagen and thus problem goes to its metabolites, although these mutagenic metabolites are unknown [8]. There is no enough evidence available to show the exact persistence of this compound in the nature but it is cleared that at room temperature, EtBr can easily dissolve in water at 10 mg/mL. Also, it should be soluble up to 20 mg/mL in water or up to 2 mg/mL in ethanol. Stock solutions of EtBr in water or phosphate buffer solution (PBS) are stable for at least two years at room temperature if protected from light [9]. It is predictable that EtBr must be very stable in the environment because it is a salt and not simply degraded. The stability of this compound reveals that how much important is to treat this compound before disposing.

A big concern with usage of EtBr is disposal of the contaminated materials to this compound. To the best of our knowledge, low concentration of EtBr has not been regulated as hazardous waste, while it is treated as hazardous waste in many labs for example *via* potassium permanganate or bleach. Although, EtBr has a potential of chemical degradation, the disposal of contaminated materials is still remained a concern. A usual way in almost all the labs is to treat contaminated materials with sodium hypochlorite (bleach) just before disposal. Unfortunately, chemical degradation of EtBr by using bleach can result in formation of compounds that are found mutagenic. However, there is no enough data to prove the mutagenic effects of degradation products. Additionally, activated charcoal and amberlite ion exchange resin are other suggested methods to remove the EtBr from solutions [10,11]. Although the application of EtBr is going to be restricted and replaced with other tags such as SYBR® products, the safety of the new substituted compounds are still in doubt and except a few data, there is no essential evidence available to confirm that they are safer than EtBr. Further investigations are recommended to compare their relative biosafety hazards.

In many developing countries, improper disposal of leftover hazardous waste materials can cause harmful chemicals if they enter into environment and contaminate both land and water. EtBr contaminated waste could leak into water supplies, if the waste is not essentially treated or easily flowed through sewer. For this reason, biosafety training is offered to anyone involved with activities at the labs that include use, handling, storage or disposal of this compound. However, more investigations are needed to clear the real carcinogenic, mutagenic and teratogenic potencies of EtBr and its degraded molecules. We recommend that if there is no choice to use better alternatives instead, do not dilute large quantities of old stocks of EtBr and not dispose into the sink [8]. Aqueous solutions (containing higher amounts than 10 ug/mL EtBr) should be filtered or deactivated. Charcoal filtration over chemical deactivation is strongly recommended. Deactivation can be confirmed by use of UV light that detects the fluorescence. There are three recognized methods for deactivation: Armour, Lunn and Sansone, and finally Quillardet and Hoffnung methods [12]. Finding more efficient treating methods and also better disposal ways are really recommended to the researchers who involved in labs using this compound. Unfortunately, in some cities, some scientific schools and research centers have been established in the city centers where there is no good management of waste material. Therefore, EtBr waste can enter into underground waters that might be used in garden, agriculture, washing or even drinking purposes. Therefore, if the way of the waste material is tracked it would not be surprising to find a link with higher rate of some human cancers. However, there are still concerns on the toxicity and safety of the compounds which are used alternative to EtBr.

## Competing interest

The authors declared that there is no conflict of interest.

## Authors’ contributions

Both authors contributed equally to the paper. Both authors read and approved the final manuscript.
